# A Lightweight Hybrid Deep Learning Model for Tuberculosis Detection from Chest X-Rays

**DOI:** 10.3390/diagnostics15243216

**Published:** 2025-12-16

**Authors:** Majdi Owda, Ahmad Abumihsan, Amani Yousef Owda, Mobarak Abumohsen

**Affiliations:** 1 Faculty of Artificial Intelligence and Data Science, UNESCO Chair in Data Science for Sustainable Development, Arab American University, Ramallah P600, Palestine; majdi.owda@aaup.edu; 2 Faculty of Artificial Intelligence and Data Science, Arab American University, Ramallah P600, Palestine; ahmaddaraghmeh413@gmail.com; 3 Department of Natural, Engineering and Technology Sciences, Arab American University, Ramallah P600, Palestine; 4 Information Technologies Group, School of Telecommunication Engineering, University of Vigo, 36310 Vigo, Spain; mobarak.abumohsen@uvigo.gal

**Keywords:** tuberculosis, chest X-ray, hybrid deep learning, GhostNet, MobileViT, feature fusion, computational cost

## Abstract

**Background/Objectives**: Tuberculosis remains a significant global health problem, particularly in resource-limited environments. Its mortality and spread can be considerably decreased by early and precise detection via chest X-ray imaging. This study introduces a novel approach based on hybrid deep learning for Tuberculosis detection from chest X-ray images. **Methods**: The introduced approach combines GhostNet, a lightweight convolutional neural network tuned for computational efficiency, and MobileViT, a transformer-based model that can capture both local spatial patterns and global contextual dependencies. Through such integration, the model attains a balanced trade-off between classification accuracy and computational efficiency. The architecture employs feature fusion, where spatial features from GhostNet and contextual representations from MobileViT are globally pooled and concatenated, which allows the model to learn discriminative and robust feature representations. **Results**: The suggested model was assessed on two publicly available chest X-ray datasets and contrasted against several cutting-edge convolutional neural network architectures. Findings showed that the introduced hybrid model surpasses individual baselines, attaining 99.52% accuracy on dataset 1 and 99.17% on dataset 2, while keeping low computational cost (7.73M parameters, 282.11M Floating Point Operations). **Conclusions**: These outcomes verify the efficacy of feature-level fusion between a convolutional neural network and transformer branches, allowing robust tuberculosis detection with low inference overhead. The model is ideal for clinical deployment and resource-constrained contexts due to its high accuracy and lightweight design.

## 1. Introduction

**Figure 1:**
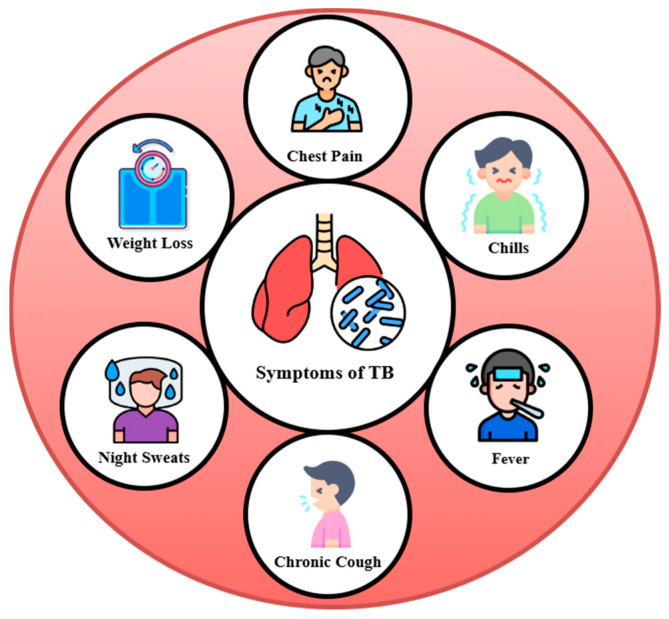
Common symptoms of TB.

Tuberculosis (TB) is a serious public health issue brought on by Mycobacterium tuberculosis. In addition to the human lungs (pulmonary TB) [1], it also has a substantial impact on the spine, brain, and kidneys (extrapulmonary TB) [2]. The most common way that TB spreads is through the air, usually by coughing and sneezing. Common signs of TB include fever, chills, night sweats, weight loss, persistent coughing, and chest pain. Figure 1 presents the symptoms of TB. Approximately 10 million people contracted the disease in 2019, of whom 1.4 million died. As the 13th most common cause of mortality worldwide, TB is the leading cause of mortality from a single infectious agent, surpassing HIV/AIDS [3]. In 2020, the number of new cases of TB decreased from the year before, making it the second most common infectious agent-related cause of death, after COVID-19. The decrease was caused by COVID-19 protocols such as social distancing and nose mask use [4]. Between 2019 and 2020, the number of new TB cases decreased from 7.1 million to 5.8 million, an 18% decrease. Although TB is treatable, a person’s health may suffer greatly if it is revealed too late. TB is common in Southeast Asia and Africa because of economic obstacles. The Eastern Mediterranean accounts for 8.3% of TB cases, the Americas for 3.0%, and Europe for 2.3%, while Southeast Asia accounts for 43%, Africa for 25%, and the Western Pacific for 18% [5].

TB is a fatal disease that can be cured with early detection and appropriate treatment [6]. Pulmonary TB is frequently detected and screened using chest X-rays (CXR) [7]. Clinical medical image analysis is generally performed by skilled radiologists, and they play a significant part in disease diagnosis and management, using chest X-ray images to reveal TB. However, there are several challenges to this method [8,9], such as that it is a time-consuming diagnostic process. Moreover, in radiograph-based disease diagnosis, subjective discrepancies are unavoidable. Furthermore, TB CXR scans are frequently misclassified into other diseases with identical radiologic characteristics. This can result in the provision of inappropriate medication to the patients, which can aggravate their condition. Additionally, there is a shortage of qualified radiologists in low-income nations, particularly in rural areas. These issues highlight how important it is to employ cutting-edge tools to help with radiological examination due to the millions of new TB cases that occur annually.

Over the past few years, the incorporation of Artificial Intelligence (AI) in medical imaging has emerged as a breakthrough solution for overcoming these limitations [10]. AI, particularly machine learning (ML) and deep learning (DL), has shown a terrific ability for automated analysis of medical images. These techniques can recognize patterns and features in images invisible to the naked eye, hence boosting diagnostic accuracy and reliability [11,12]. ML techniques, with their power to lighten the workload of clinicians, diminish the possibilities of human mistakes and boost diagnostic precision. By integrating these systems into routine clinical practice, medical professionals can get precise and dependable outcomes, which consequently enhance patient outcomes [13]. However, conventional ML methods need manual feature extraction, which is time-consuming and cannot extract complicated features [14]. DL has become a precious tool since it is able to extract automatically complex and hierarchical features from medical images without the need for manual feature engineering [14]. Convolutional neural network (CNN) is a common DL technique used in medical image analysis due to its ability to extract spatial representations from medical scans, which overcomes the limitation of hand-crafted feature engineering associated with traditional ML models [15,16].

Although CNN approaches have shown excellent results in detecting tuberculosis in numerous studies [17,18,19,20,21], they still have certain limitations. CNNs are unable to efficiently extract global information [22]. CNNs are computationally costly due to their depth structure and large number of parameters; therefore, they are less appropriate for low-resource environments [23].

In general, DL algorithms have been extensively used in medical image analysis. For instance, the research in [24] introduced a two-dimensional multiscale symbolic dynamic entropy model to establish a computer tool sensitive to subtle pattern fluctuations associated with pulmonary tuberculosis and noise robustness for analyzing CXR images. Research in [25] presented a lightweight convolutional neural network (CovidxNet-CT) for the classification of COVID-19 from chest CT images. Researchers in [26] leveraged the Segment Anything Model for developing a classification TB model from CXR images. Moreover, they used two lightweight attention mechanisms, Improved Linear Attention and Chunk-Wise Attention Block, to reduce computational complexity without affecting accuracy.

In computer vision tasks, Vision Transformers (ViTs), which feature the self-attention phenomenon, have recently replaced CNNs [27]. Moreover, ViTs have demonstrated their efficiency in medical image detection, classification, and segmentation. In medical image analysis, ViTs provide a number of benefits [28,29]. The first is global feature extraction; ViTs process the entire image as a series of patches, enabling them to capture long-range relationships, in contrast to CNNs, which rely on localized filters. Another benefit is better generalization on varied data; ViTs perform better than CNNs in image variability since they are self-attention-based, and this minimizes bias towards certain features. However, as much as they are advantageous, ViTs are even more computationally expensive compared to CNNs due to the high-dimensional self-attention mechanism. For many real-time and low-power medical applications, this renders the use of ViTs unfeasible [30].

To strike a balance between accuracy and computing efficacy, we propose a hybrid model based on GhostNet and MobileViT for TB detection from chest X-rays. The model is developed to maintain high accuracy while reducing computational expense; thus, this model can be deployed in resource-limited settings. GhostNet is an extremely effective CNN architecture that maintains deep networks’ capacity for feature extraction while lowering computational overhead [31]. MobileViT is a light-weight hybrid model comprising CNN and Vision Transformer (ViT) blocks. In contrast to standard CNNs, which depend on local receptive fields, MobileViT leverages self-attention mechanisms for capturing long-range dependencies while maintaining computational efficiency [32].

ML and DL techniques have been extensively employed in numerous studies for TB detection from chest X-ray images [17,18,19,20,21,33,34,35,36]. Although these methods have demonstrated promise, accurately detecting TB considering the computational complexity remains a formidable challenge. Many previous studies utilized relatively small datasets, thus inhibiting the model from effectively generalizing to different populations and clinical settings. Many studies relied on handcrafted feature extraction techniques, which struggle to learn and extract complex hierarchical patterns present in chest X-ray images. Several existing approaches often rely on single models, which may limit the model’s ability to capture a variety and complementary feature representations. Many approaches attained excellent accuracy, but their high computational complexity can limit their applicability in low-resource environments. Notable research gaps that restrict the efficacy and generalizability of current TB detection techniques are shown by our analysis of the recent literature. The following is a summary of these gaps:

• Limited Dataset Size: The development of a robust TB detection model highly relies on the availability of sufficiently large and diverse datasets. However, some of the existing research utilized relatively small datasets, thus inhibiting the model from effectively generalizing to different populations and clinical settings.
• Inadequate Handling of Complex Patterns: Many conventional methods rely on handcrafted feature extraction techniques, which struggle to learn and extract complex hierarchical patterns present in chest X-ray images. This restricts their ability to make fine distinctions between TB and non-TB conditions.
• Single-Model Dependency: A significant portion of earlier research is based on a single deep-learning model, which may limit the model’s ability to capture diverse and complementary feature representations. Such reliance can negatively impact detection performance.
• High Computational Cost: Although some cutting-edge techniques attained excellent accuracy, their high computational complexity can limit their real-time applicability and deployment viability in environments with limited resources.

These gaps have motivated us to develop a novel hybrid deep-learning model for tuberculosis detection from chest X-ray images. The following are the main contributions of this work:

• Development of a Hybrid Deep Learning Model: A novel hybrid deep learning approach is introduced by combining GhostNet, a light-weight CNN, with MobileViT, a transformer-based model. The combination leverages the power of both local feature extraction (CNN) and global context modeling (transformers) for more accurate TB classification.
• Feature Fusion Strategy: An effective feature-level fusion mechanism is developed, wherein feature maps pooled globally from both GhostNet and MobileViT are concatenated and passed through a joint classification head.
• Balanced Trade-Off Between Efficiency and Performance: The introduced model attains high diagnostic accuracy (up to 99.52% accuracy) while preserving computational efficiency (just 7.73 million parameters and 282.11M Floating Point Operations (FLOPs), superior to most state-of-the-art models.
• Thorough Assessment on Two Datasets: This study employs two independently sourced chest X-ray datasets—dataset 1, with a total of 7000 images (3500 TB and 3500 normal), and dataset 2, with 1600 images (800 TB and 800 normal). Each dataset was randomly divided into training (70%), validation (15%), and testing (15%) subsets. All performance metrics reported in this study are based exclusively on the held-out test subsets, ensuring an unbiased evaluation of the model’s generalization capability across different imaging distributions.
• Five-fold cross-validation: A comprehensive 5-fold cross-validation evaluation was conducted to ensure statistical reliability and demonstrate consistent model generalization across different partitions of the dataset.
• Mathematical Formalization of the Hybrid Network: A comprehensive mathematical modeling of the suggested hybrid architecture is presented.

It is worth mentioning that the selection of GhostNet and MobileViT-XS was impelled by the need to balance high diagnostic accuracy with minimal computational cost, which is considered an essential issue for TB screening in resource-constrained clinical settings. GhostNet was picked from among other lightweight CNNs, such as MobileNetV3 or EfficientNet-Lite, because it makes use of ghost modules, generating intrinsic and cheap feature maps with significantly fewer parameters and FLOPs while maintaining the representational capacity [31,37]. MobileViT-XS was chosen as the transformer branch due to its hybrid design, which incorporates convolutional inductive biases with transformer-based global attention mechanisms into a lightweight and mobile-friendly concept. Compared to heavier vision transformers (e.g., ViT, DeiT), MobileViT achieves global context modeling with substantially fewer parameters [32,38].

The remaining part of the paper is organized as follows. Section 2 presents the related works of this research. Section 3 provides the methodology of this research, including data collection, the proposed model, and mathematical modeling of the proposed hybrid network. The discussions and findings are explained in Section 4. The significant conclusions of this research are finally presented in Section 5.

## 2. Literature Review

### 2.1. Machine Learning

The authors in [33,34,35,36] employed ML algorithms for the detection of TB from CXR images. Authors in [33] suggested a TB detection system from chest X-rays based on ML algorithms. The Inception V-3 algorithm has been utilized for the feature extraction process and four ML models were used for the classification process: support vector machine (SVM), K-Nearest Neighbors (KNN), Random Forest (RF), and neural network (NN). The findings proved that the NN yielded the best efficiency compared with its counterparts. In [34], the authors developed a technique for the detection of TB from CXR images based on handcrafted geometrical features (HGF), first-order statistical features (FOSF), and SVM. Utilizing two publicly available datasets, they obtained encouraging results with accuracy ranging from 95.6% to 99.4%. In [35], the authors suggested an automated system for TB identification in chest X-rays. They used several handcrafted feature extraction methods like Histogram of Oriented Gradients (HOG), Local Binary Patterns (LBP), Gradient Magnitude Histograms (GM), Shape Descriptors (SD), and Intensity Histograms (IH) for the feature extraction process and SVM for the classification. Their technique was tested on two public datasets and attained a good accuracy. The study of [36] introduced a technique for identifying TB disease based on KNN and a handcrafted feature extraction method (Hog Feature Extraction). The presented method was assessed using one public dataset, and good results were obtained.

### 2.2. Deep Learning

Convolutional neural network (CNN) is a terrific model and has been employed in many studies for the detection of TB from CXR scans [17,18,19,20,21]. The study in [17] investigated the use of a deep CNN for the identification of TB in chest X-rays. It highlighted the effectiveness of lung segmentation and both lossless and lossy data augmentation in improving training on a small, imbalanced dataset. While segmentation and lossless augmentation reduced overfitting, loss augmentation decreased validation accuracy. The authors in [18] introduced a simple CNN for TB detection from chest X-ray images; the suggested technique aimed to reduce computational complexity. The proposed method has been assessed on three public datasets and attained good efficiency. The authors in [19] employed a Bayesian convolutional neural network (B-CNN) for TB identification from CXR images. They evaluated their approach on two public datasets and attained an accuracy of 96.42% on the Montgomery dataset and 86.46% on the Shenzhen dataset. The authors in [20] presented a DL-based approach for TB detection using lung segmentation and a multicategory lesion detection approach. A scalable pyramid structure was incorporated into Faster RCNN with reinforcement learning to improve small-lesion detection. They evaluated their approach on multiple public datasets and attained an accuracy of 0.926% on the Montgomery dataset and 0.902% on the Shenzhen dataset. In [21], the authors introduced a CNN-based classification technique to classify multiple diseases (tuberculosis, pneumonia, and COVID-19) from chest X-ray images. They evaluated their technique on public datasets and attained phenomenal results.

The authors in [39,40,41,42,43,44,45,46,47] employed pre-trained CNN algorithms in their approach. The authors in [39] assessed the performance of nine pre-trained CNN models (ResNet18, ResNet50, ResNet101, CheXNet, InceptionV3, VGG19, DenseNet201, SqueezeNet, and MobileNet) for TB detection from chest X-ray images, with and without the segmentation process. Employing four public datasets, the study proved that the segmentation process significantly boosted the classification process, where the DenseNet201 attained the best outcomes compared with its counterparts. The authors in [40] presented a technique for TB detection based on a lightweight MobileNet; the suggested model was assessed on three public datasets and achieved terrific results. In [41], the authors presented a modified DenseNet121 model for TB detection from chest X-ray images; they assessed their method on two public datasets and attained remarkable outcomes. In [42], three CNN models (AlexNet, VGG-16, CapsNet) have been utilized for TB classification from chest X-ray images. Three datasets have been employed to evaluate the CNN models. CapsNet demonstrated better robustness to affine transformations compared to AlexNet and VGG-16, but still had limitations, achieving an accuracy of 80.06%. In [43], the authors suggested a method based on VGG16 for TB detection in CXR images without requiring segmentation. Their findings demonstrated that data augmentation increased the accuracy to 81.25%. Moreover, CNNs offer a more straightforward yet efficient method than conventional segmentation-based models since they can function well without requiring much pre-processing. The authors in [44] developed a CBAMWDnet model for detecting TB in CXR images. They employed the convolutional block attention module (CBAM) and the wide dense net (WDnet) and evaluated their approach using three public datasets. The suggested system attained an accuracy of 98.80%, sensitivity of 94.28%, precision of 98.50%, specificity of 95.7%, and F1-score of 96.35%. In [45], the authors proposed a ResNet-fused External Attention Network (ResfEANet) model for classifying TB from CXR images. The suggested model is a shallower design than ResNet-50 since it contains fewer residual blocks. Although this method lowers computational costs, it may make it more difficult for the model to identify intricate patterns in extremely large or varied datasets. Generalizability for more complex tasks may be impacted by this. The study in [46], introduced an investigation of the impacts of using image enhancement techniques on the performance of DL techniques for TB detection. The enhancement techniques used in the study involve Unsharp Masking (UM) and High-Frequency Emphasis Filtering; the DL models used are ResNet-50, ResNet-18, and EfficientNet-B4. The suggested method demonstrated a considerable improvement in classification performance with an accuracy of 89.92% and an AUC of 94.8%. A comparative study was conducted in [47] to assess the performance of five pre-trained DL models (AlexNet, VGG-16, VGG-19, Xception, ResNet-50) for TB detection from CXR images. Four datasets have been utilized. The findings demonstrated that AlexNet and VGG-16 are more efficient than their counterparts.

Various researchers have suggested a hybrid technique for TB detection from CXR images [48,49,50,51]. In [48], the authors introduced a hybrid technique for TB detection from CXR images. They used MobileNet for feature extraction and the Artificial Ecosystem-based Optimization (AEO) algorithm for feature selection. Their methodology was tested on two publicly available datasets, one specifically for TB and the other for pneumonia, attaining 90.2% accuracy on dataset 1 and 94.1% accuracy on dataset 2. A Convolutional Neural Network–Long Short-Term Memory (CNN-LSTM) hybrid model was introduced in the work [49] to categorize pulmonary illnesses from chest X-ray scans. Among the pulmonary illnesses included were COVID-19, pneumonia, and tuberculosis. When the proposed model was compared to the CNN and CNN–Bidirectional LSTM baseline models, it performed better and achieved an accuracy of 96.24%. The authors introduced the hybrid ResNet–SVM model for TB detection from chest X-rays in [50]. They evaluated their strategy using two publicly available datasets and attained good outcomes. The study in [51] suggested a hybrid DL model for TB detection. The introduced hybrid model blended three DL models: CNN to extract spatial features from X-ray images; Recurrent Neural Networks (RNN), for sequential analysis of radiology reports; and Artificial Neural Networks (ANN), to refine the features and provide a final classification. It was assessed based on three public datasets and achieved remarkable outcomes.

Several studies have suggested an ensemble technique in their approach [52,53,54]. The authors in [52] proposed a TB detection system based on an ensemble method. They employed three techniques: AlexNet, GoogleNet, and ResNet. Four public datasets were utilized. Their approach attained an accuracy of 88.24%. In [53], the authors presented a multiple-instance learning approach for TB recognition from CXR images; they employed Xception and DenseNet for feature extraction, and they suggested a stacked ensemble classifier combining multiple conventional ML algorithms (Logistic Regression (LR), AdaBoost, Decision Tree (DT), RF, and SVM). The suggested approach was evaluated on two public datasets and yielded terrific results. The study in [54] presented a stacked ensemble technique for TB detection from CXR images; the suggested technique combined several handcrafted feature extraction methods (Histogram of Oriented Gradients (HOG), and Speeded-Up Robust Features (SURF)) and several pre-trained CNN algorithms (AlexNet, VGG-16, GoogLeNet, ResNet-50) and SVM for classification. The study assessed the technique on four public datasets; its findings proved that ensemble learning enhances classification efficiency.

Table 1 lists the prior research, including the dataset, methodology, findings, and limitations for each study.

Although the methods used in the existing studies have demonstrated promise, there remain significant limitations. Many previous studies utilized relatively small datasets, which restricts the model from effectively generalizing to different populations and clinical settings. Moreover, many studies relied on handcrafted feature extraction techniques, which struggle to learn and extract complex hierarchical patterns present in chest X-ray images. Furthermore, several existing approaches often rely on single models, which may limit the model’s ability to capture a variety and complementary feature representations. While many approaches attained excellent accuracy, their high computational complexity can limit their real-time applicability or environments with constrained resources.

To bridge these gaps, we introduced a novel hybrid model that has been developed and validated using large-scale datasets. The proposed model combines GhostNet, a lightweight CNN, with MobileViT, a transformer-based model, hence combining efficient local feature extraction with global contextual understanding. This fusion leverages the strengths of both networks and allows the model to learn richer, more discriminative representations. It is worth mentioning that the model attains a balanced trade-off between performance and efficiency, obtaining high diagnostic accuracy while preserving computational efficiency suitable for real-world deployment.

## 3. Methodology

**Figure 2:**
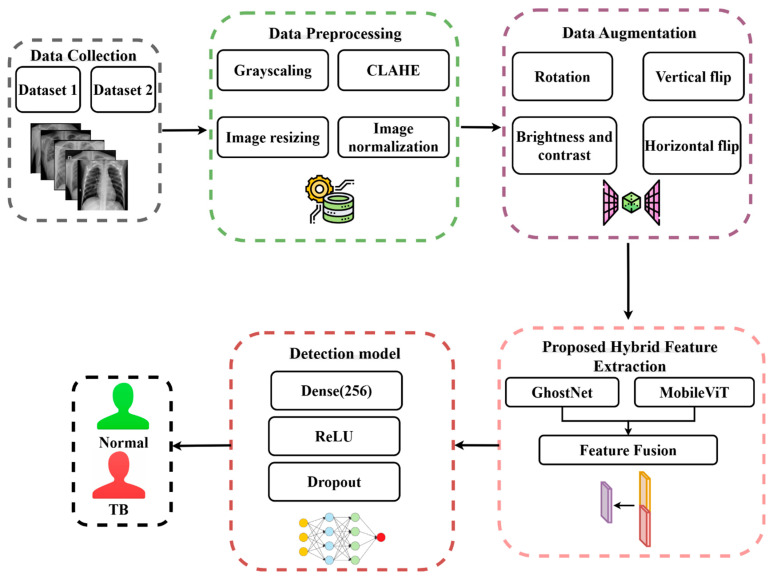
The methodology used in this research.

The methodology employed in this work is depicted in Figure 2 and it includes a series of steps: (1) data collection—shows the datasets utilized in this study; (2) data pre-processing—it explains the preprocessing methods that have been employed; (3) data augmentation—it illustrates image augmentation techniques that have been used on the dataset; (4) feature extraction—presents the pre-trained models that have been employed, GhostNet and MobileViT, as well as the proposed feature fusion and the classification process.

### 3.1. Data Collection

This section presents the datasets used in this study, namely dataset 1 and dataset 2. Dataset 1 comprises public data released in 2020 by Rahman et al. [39], called the CXR database, and contains 7000 scans. Dataset 2 also comprises public data, called TBX11K [55], and involves 1600 images.

#### 3.1.1. Dataset 1

This dataset is called the “CXR database”, originally released in 2020 by Rahman et al. [39]. It comprises 7000 CXR images, which are equally divided into healthy individuals and persons with tuberculosis (3500 normal cases and 3500 TB cases). The CXR database has been aggregated from four datasets:

• The National Library of Medicine (NLM) Dataset [56], which contains images from two lung X-ray datasets: The Shenzhen, China, dataset (336 TB positive, 326 healthy, 3000 × 3000 pixels) and the Montgomery County dataset (58 TB positive, 80 healthy, 4020 × 4892 pixels).
• Belarus Dataset [57]: Various organizations under the Republic of Belarus’s Ministry of Health put together the dataset. It includes the CXRs of 306 TB patients; each scan has a resolution of 2248 × 2248 pixels.
• NIAID TB Portal Program Dataset [58]: A total of 2800 TB-positive CXR images from roughly 3087 cases are included in the NIAID TB portal program dataset and were gathered from seven different countries.
• RSNA Pneumonia Detection Challenge Dataset [59]: Out of 10,000 scans, Rahman et al. [39] chose 3094 scans of healthy people.

In total, the utilized dataset involves 3500 TB-positive scans (306 from Belarus, 2800 from NIAID, and 394 from NLM) and 3500 healthy scans (406 from NLM and 3094 from RSNA).

#### 3.1.2. Dataset 2

This dataset is called the TBX11K dataset [55] and involves three classes (TB, Healthy, and Sick but Not TB). The collection comprises 11,200 X-ray scans for the three classes, but these classes are not balanced. In this research, we randomly selected 1600 images for healthy people and people with TB (800 for normal cases and 800 for TB cases). Each image has a pixel size of 512. Table 2 shows the summary of the datasets used in this work.

### 3.2. Data Preprocessing

Before the images can be used for training models and inference, they need to be preprocessed. This can make model training faster and also accelerate inference [60,61]. The image preprocessing techniques used in this study are as follows:

• Grayscaling: This refers to the conversion of RGB images to grayscale.
• Image resizing: The grayscale image was rescaled to a particular size, that is 128 × 128 pixels.
• Contrast Limited Adaptive Histogram Equalization (CLAHE): This improves the contrast of the image, making the tuberculosis area more pronounced and clearer.
• Image normalization: Normalization adapts the intensity range of individual pixels, typically to make the pixel values of an image more consistent and uniform to the human eye.

**Figure 3:**
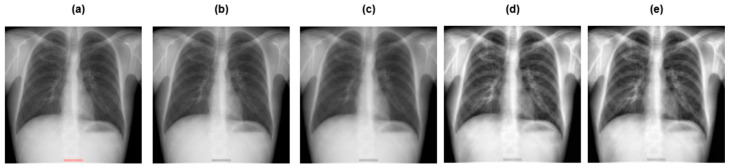
Preprocessing techniques applied to dataset 1: (**a**) original image; (**b**) grayscaling; (**c**) image resizing; (**d**) CLAHE; (**e**) image normalization.

**Figure 4:**
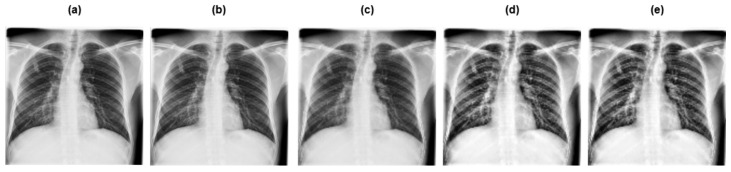
Preprocessing techniques applied to dataset 2: (**a**) original image; (**b**) grayscaling; (**c**) image resizing; (**d**) CLAHE; (**e**) image normalization.

Figure 3 and Figure 4 depict the pre-processing techniques applied to dataset 1 and dataset 2, respectively.

### 3.3. Data Augmentation

**Figure 5:**
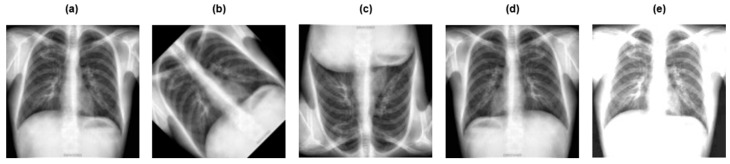
Various image augmentation methods employed on dataset 1: (**a**) original image; (**b**) 45-degree rotation; (**c**) vertical flip; (**d**) horizontal flip; (**e**) brightness and contrast adjustments.

**Figure 6:**
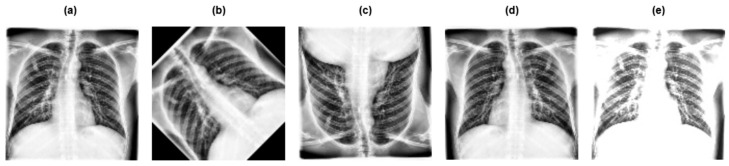
Various image augmentation methods employed on dataset 2: (**a**) original image; (**b**) 45-degree rotation; (**c**) vertical flip; (**d**) horizontal flip; (**e**) brightness and contrast adjustments.

Data augmentation is a technique used to create new data points from the available data in order to artificially boost the size of the dataset. Augmented data is the data that has been transformed minimally from the original version using geometric transformations such as flipping vertically and horizontally, rotating, and brightness and contrast adjustments in order to enhance the variety of the training set [14]. Figure 5 and Figure 6 show different image augmentation techniques used on dataset 1 and dataset 2, respectively.

### 3.4. Feature Extraction

The performance of an ML classifier largely relies on the input feature vector. Hence, developing an algorithm with the capability to extract significant and distinctive features from TB CXR images is critical to obtaining accurate TB classification.

#### 3.4.1. GhostNet

A comprehensive grasp of the input data is ensured by the feature maps of well-trained deep neural networks, which often involve copious and even redundant information. A deep learning network’s redundancy in feature maps could be significant. The GhostNet architecture prioritizes computational cost effectiveness while maintaining redundant feature maps. Han et al. introduced the GhostNet lightweight network in 2020. It is a CNN that is lightweight and built by stacking Ghost Bottlenecks, which consist of Ghost modules [31]. It has been demonstrated that GhostNet is more accurate and can characterize more features with fewer computations than other lightweight network models, like the MobileNet family [62,63] and the ShuffleNet family [64,65].

**Figure 7:**
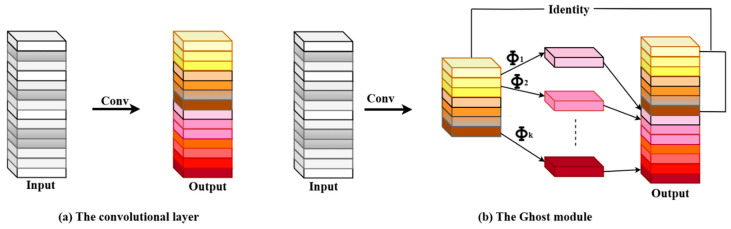
Schematic of various convolution modes: (**a**) traditional convolution; (**b**) Ghost module.

The Ghost module creates a ghost feature map by using the Φ as the linear operation, as opposed to the original convolutional layer. Figure 7 illustrates the difference between the convolution modes for the traditional convolution and the Ghost module.

Equation (1) represents the feature map computation in the original convolutional layer:

(1) γ=χ×f+β

where γ represents the feature map output and χ represents the input data, χ $\in \;R^{h\times w\times c}$. Here, c stands for the input channel numbers and h and w for the height and weight of the input data, respectively. In the layer where f ∈ $R^{c\times k\times k\times n}$, f denotes the convolutional filter, n is the number of feature maps, and k × k is the kernel size of the convolutional filter.

This differs from the Ghost module, which uses the operation of Φ, represented by Equation (2), to generate output feature maps:

(2) $$\gamma_{a,b}^{\prime }=\emptyset_{a,b}(\gamma_{a}^{\prime })$$

where $\gamma_{a,b}^{\prime }$ stands for the output ghost feature maps, $\gamma_{a}^{\prime }$ is the a-th basic feature map, and $\emptyset_{a,b}$ is the linear operation that creates the b-th of $\gamma_{a,b}^{\prime }$ ghost feature maps.

**Figure 8:**
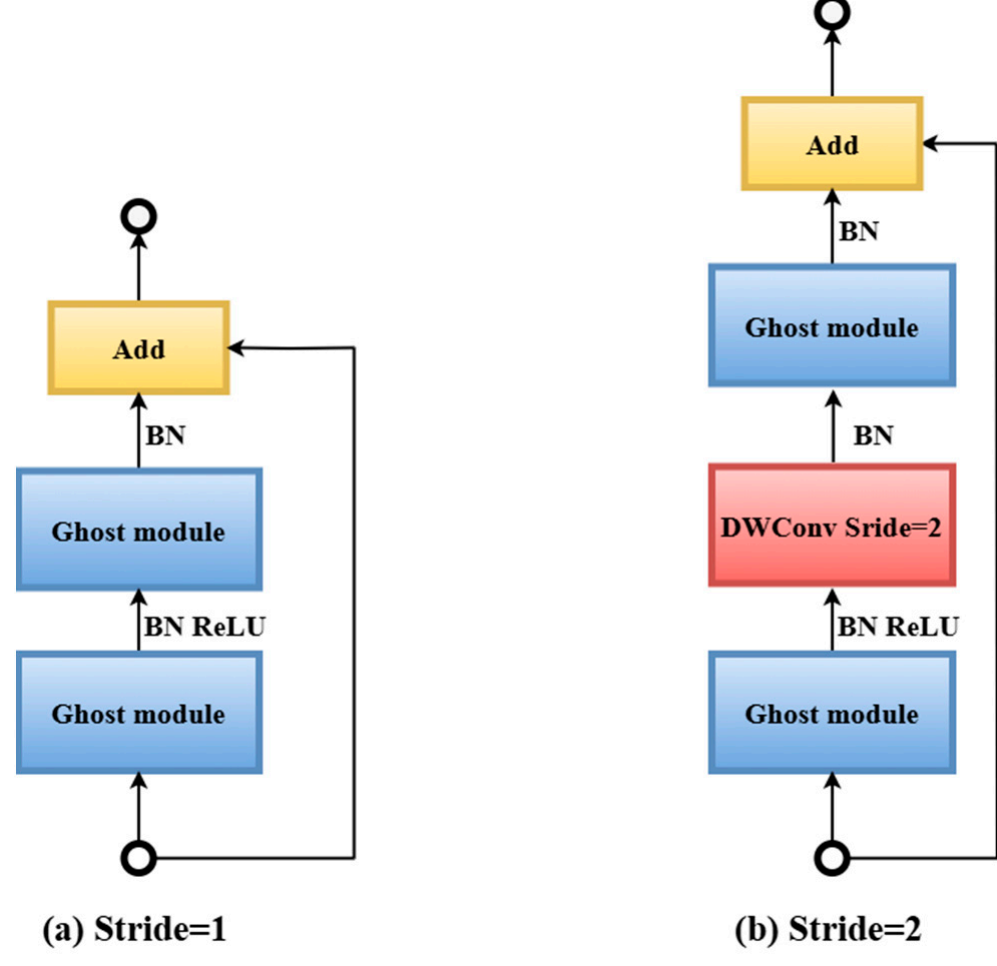
The GhostNet bottleneck map. BN—Batch Normalization; DWConv—Depthwise Convolution.

GhostNet is mostly made up of the Ghost bottleneck layer, which has multiple Ghost modules. Figure 8a depicts the Ghost bottleneck with stride 1, which is made up of the rectified linear units (ReLU) activation function, batch normalization (BN), and Ghost modules. In Figure 8a, the first Ghost module (down) is used to reduce the dimensionality, while the Ghost module (up) is used to increase the dimensionality. Hence, the input feature dimension is definitely the same as the output one, and they can be added to each other. The Ghost bottleneck with stride 2 is shown in Figure 8b. In contrast to the Ghost bottleneck with step 1, a BN layer and Depthwise convolution have been added to reduce the model’s calculation amount and number of parameters, improving the model’s lightweight level.

#### 3.4.2. MobileViT

MobileViT is a hybrid lightweight model that integrates CNN and Vision Transformer (ViT) to attain high computational efficacy with robust efficiency in visual jobs [32]. In a standard Vision Transformer (ViT), the image is split into patches, each patch is transformed into an n-dimensional feature vector, a classification token (CLS token) is prepended, and positional embeddings are added. This sequence is then fed into the Multi-Head Attention module, producing contextualized embeddings; the final CLS token embedding is used as the overall feature representation.

MobileViT captures fine-grained spatial information like edges and textures by employing convolutional layers to extract local features. These features are processed by Transformer blocks, which effectively comprehend complicated image patterns by capturing global context and long-range interdependence. The main benefit of MobileViT is its efficiency. The model is perfect for deployment in resource-constrained devices like embedded systems and mobile phones since it lowers computing costs by mixing convolutional processes with Transformer layers, which is characterized against the typical ViT models [32]. The model can capture both local features and global context because of its hybrid design, which also makes multi-scale feature extraction possible, thus being powerful in many applications like image.

**Figure 9:**
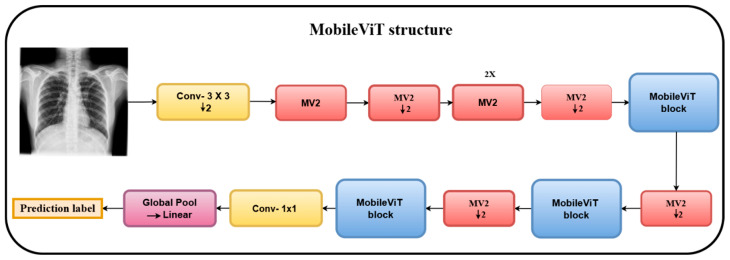
MobileViT structure. A stride of 2 is shown by the MV2 structure with a downward arrow, signifying downsampling.

Figure 9 depicts the MobileViT structure, which is comprised primarily of fully connected layers, global pooling, Mobile-ViT blocks, MV2 layers (MobileNetV2 inverted residual blocks), and depthwise convolution layers [32].

**Figure 10:**
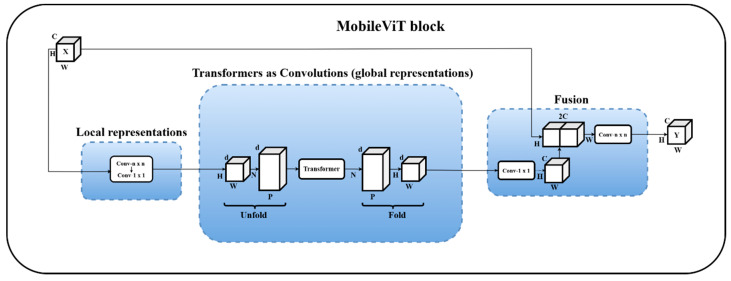
MobileViT block. Data size is denoted by C, H, W, d, N, and P, while input and output are denoted by X and Y, respectively.

The MobileViT block, which is the most crucial and essential part, starts its workflow by applying a 3 × 3 convolution layer for local feature representation. Following that, a 1 × 1 convolution layer is employed to tune the channel number. Then, global feature modeling is performed using the unfold, Transformer, and fold structures. Then, a 1 × 1 convolution layer is utilized to recover the number of channels to the initial size. The output is then produced by a 3 × 3 convolution layer for feature fusion after a shortcut branch is employed to connect the feature map along the channel dimension with the original input feature map [32]. Figure 10 shows the MobileViT block.

Replacing local modeling of convolution with global modeling via Transformer is one of the most crucial phases in the MobileViT workflow. To achieve this, Unfold and Fold operations must be conducted to reformat the data in a form required for calculating self-attention [66,67].

**Figure 11:**
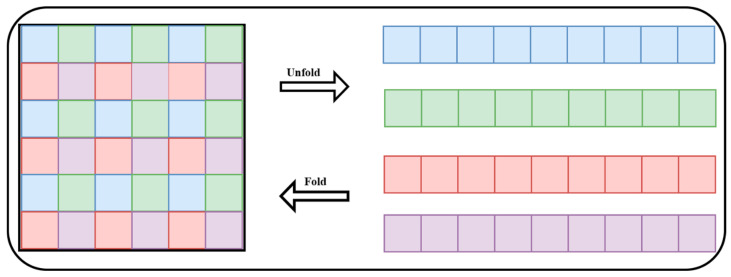
Fold and unfold procedures.

To decline computing complexity, just tokens of the same hue are taken into consideration for the self-attention calculation within the MobileViT block, as depicted in Figure 11. Tokens of the same color are flattened into a sequence by the Unfold procedure, which enables each sequence to be computed in parallel using the original self-attention mechanism. The sequences are then folded back into the original feature map using the Fold procedure. Using this method, MobileViT efficaciously combines local and global features, eventually conducting feature fusion through a convolutional layer to provide the output. This architecture maintains great performance while lowering computational loads. Figure 11 shows the Fold and Unfold procedures.

#### 3.4.3. Proposed Model

**Figure 12:**
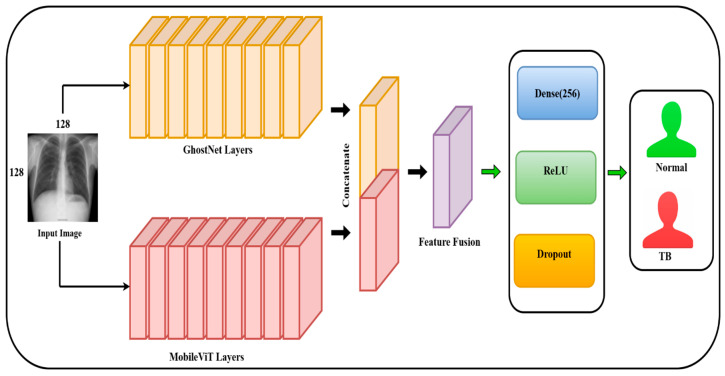
Proposed hybrid deep learning model.

In this paper, we propose a hybrid deep learning model that synergizes the strengths of convolutional and transformer models for improving the classification performance of TB from chest X-ray images. The model uses two lightweight yet effective backbones: GhostNet, a computationally efficient convolutional neural network, and MobileViT-XS, a transformer-based model with the capability to extract both local spatial information and global contextual patterns. The novelty of our approach lies in the combination of GhostNet and MobileViT-XS with Batch Normalization (BN) fusion, identity-based normalization removal, and a compact feature-fusion strategy. Figure 12 depicts the proposed hybrid deep-learning model.

The hybrid model is developed to capture diverse and complementary features by passing the chest X-ray images through two parallel paths:

• GhostNet Path: GhostNet uses a series of ghost modules to generate more feature maps with fewer computations. It concentrates on capturing spatial and texture-level patterns that are generally present in medical imaging data.
• MobileViT Path: MobileViT leverages the inductive bias of CNNs combined with the long-range feature modeling capacity of transformers. This enables the network to efficiently capture fine-grained details as well as coarse contextual clues from the input images.

During model construction, all Batch Normalization layers have been removed in both GhostNet and MobileViT to improve efficiency and decrease redundancy. Instead of discarding them, each BN layer is mathematically merged into its corresponding convolution layer through a custom BN–Conv fusion routine. Once fusion is performed, Identity mappings are used in place of all Batch Normalization layers in both GhostNet and MobileViT, which implies a BatchNorm-free forward pass. This strategy reduces the inference cost while retaining the normalization effects learned during pretraining.

Both backbones are initiated using ImageNet pre-trained weights to utilize transfer learning benefits. After the input image is processed separately by the GhostNet and MobileViT backbones, Global Average Pooling (GAP) is applied to the final feature maps of the two branches to generate two fixed-length embedding vectors. The two vectors are concatenated to form a uniform representation that is able to efficiently capture both the local spatial features and the global contextual information.

The fused feature vector is then passed through a lightweight classification head, which consists of a fully connected layer of 256 neurons, a ReLU activation function, and a Dropout layer of rate 50% to decrease overfitting. Finally, a dense layer of 2 output neurons is used to perform binary classification, discriminating between TB-positive and normal instances. The entire model is trained end-to-end using the cross-entropy loss function and optimized using the Adam optimizer to ensure an optimum trade-off between classification performance and computational efficacy.

#### 3.4.4. Mathematical Modeling of the Proposed Hybrid Network

This section describes the mathematical formulation of the suggested hybrid deep learning model incorporating GhostNet and MobileViT-XS for tuberculosis detection from chest X-ray images.

1. Input and Pre-processing

The input is the chest X-ray image ($X_{raw}$), the following is performed on the raw image:

• Resized to 128 × 128 and converted to grayscale: $X_{raw}\in$ $R^{H\times W}$, where H = W = 128 (grayscale image).
• Enhanced using CLAHE (Contrast Limited Adaptive Histogram Equalization): $X_{clahe}=CLAHE(X_{raw})$.
• Normalized and converted to a 3-channel image for compatibility with pre-trained models [68].

(3) $$X_{rgb}=Stack(X_{clahe},\;X_{clahe},\;X_{clahe})\in R^{128\times 128\times 3}$$

(4) $$X_{norm}=\frac{X_{rgb}-µ}{\sigma },\;µ=0.5,\;\sigma =0.5$$

(5) $$X_{norm}=\frac{X_{rgb}-0.5}{0.5}\in R^{128\times 128\times 3}$$

Here, µ is the mean and σ is the standard deviation.

2. GhostNet Branch

Let $f_{g}$() represent the GhostNet backbone [31], which extracts convolutional features. After feature extraction and global average pooling (GAP), the output feature vector is

(6) $$F_{g}=GAP(f_{g}\left(X_{norm}\right))\in R^{d_{g}}$$

(7) $$F_{g}=GAP(f_{g}\left(X_{norm}\right))\in R^{1280}$$

where $F_{g}$ is the compact representation of local texture features learned by GhostNet, and $d_{g}$ = 1280 denotes the feature dimension of GhostNet after GAP.

3. MobileViT Branch

Let $f_{m}$() represent the MobileViT-XS backbone [32], which integrates CNN and transformer layers for extracting both local and global representations. After global pooling,

(8) $$F_{m}=GAP(f_{m}\left(X_{norm}\right))\in R^{d_{m}}$$

(9) $$F_{m}=GAP(f_{m}\left(X_{norm}\right))\in R^{6144}$$

where $F_{m}$ captures global dependencies and semantic context, and $d_{m}$ = 6144 is the dimensionality of MobileViT’s output features.

4. Feature Fusion

The final representations from both backbones are concatenated to form a unified feature vector:

(10) $$F_{fused}=[F_{g}⊕F_{m}]\in R^{d_{g}+d_{m}}$$

(11) $$F_{fused}=[F_{g}⊕F_{m}]\in R^{7424}$$

where

• ⊕ denotes the concatenation operation;
• $F_{fused}$ ∈ $R^{1280+6144}$ = $R^{7424}$.

5. Classification Head

The fused vector is passed through a fully connected classification head:

1. Fully Connected Layer with ReLU:
  (12) $$h_{1}=ReLU\left(W_{1}F_{fused}+b_{1}\right),W_{1}\in R^{256\times 7424}$$
  where $W_{1}$ is the weight matrix of the first fully connected layer and $b_{1}$ is the bias vector.
2. Dropout for regularization:
  (13) $$h_{2}=Dropout\left(h_{1},p=0.5\right)$$
3. Final classification layer:
  (14) $$\hat{y}=Softmax\left(W_{2}h_{2}+b_{2}\right),W_{2}\in R^{2\times 256}$$
  where $\hat{y}\in R^{2}$ is the predicted probability distribution over the two classes (normal, and TB), $W_{2}$ is the weight matrix of the final output layer, and $b_{2}$ is the bias vector for the final layer.

6. Optimization Objective

• The model is trained using the cross-entropy loss [69]:
  (15) $$ℒ=-\sum_{i=1}^{N}y_{i}log({\hat{y}}_{i})$$
  where $y_{i}$ is the ground-truth label and ${\hat{y}}_{i}$ is the predicted probability from the softmax output.
• The model is optimized using the Adam optimizer [70] with a learning rate of 0.001.
  (16) $$\theta \;\leftarrow \;\theta -\alpha .\;{▽}_{\theta }\;ℒ,\;\mathrm{where}\;\alpha =0.001$$
  where θ is the model parameters, ${▽}_{\theta }ℒ$ is the gradient of the loss function ℒ with respect to the parameters θ, and α is the learning rate.

7. The final pipeline of the suggested approach can be described as shown in Equation (17):
  (17) $$\hat{y}=Softmax\;\left(W_{2}\;Dropout\;(ReLU\;\left(W_{1}\cdot [GAP\left(f_{g}\left(X_{norm}\right)\right)⊕\;GAP\left(f_{m}\left(X_{norm}\right)\right)]+b_{1}\right))+b_{2}\right)$$

This hybrid model leverages both CNN and transformer-based representations, aiming for strong TB detection performance with minimal computational overhead.

## 4. Results and Discussion

### 4.1. Performance Metrics

For assessing the performance of our proposed method, we applied well-known evaluation metrics frequently used in ML and DL tasks [71,72,73], involving accuracy, precision, recall, and F1-score. Each formula is illustrated in Equations (18)–(21):

(18) $$Accuracy=\frac{TP+TN}{TP+TN+FP+FN}$$

(19) $$Precision=\frac{TP}{TP+FP}$$

(20) $$Recall=\frac{FP}{TN+FP}$$

(21) $$F1\;Measure=2\times (\frac{Precision\times Recall}{Precision+Recall})$$

where FP stands for False Positive, FN for False Negative, TP for True Positive, and TN for True Negative.

In addition to the predictive evaluation metrics, we evaluate the computational efficiency of the suggested model using the number of parameters and FLOPs (Floating Point Operations). The entire amount of learnable weights in a neural network is referred to as parameters. A more compact model is usually indicated by fewer parameters, which might be advantageous for deployment in devices with constrained memory or processing power. The total number of computations needed to complete a single forward pass through the network is represented by FLOPs. It is essential for assessing energy efficiency and real-time applicability and acts as a gauge of the model’s computing cost [74,75,76,77].

We employed the THOP profiling library in PyTorch PyTorch 2.6.0 to calculate he number of parameters and FLOPs for all models. FLOPs have been measured by performing a single forward pass through the network using an input tensor of size 128 × 128 × 3, which matches the configuration utilized while training and evaluation. THOP works by internally accumulating the computational cost of each layer to compute the total FLOPs value, and Parameter counts are directly acquired from the model’s learnable weights [78,79].

### 4.2. Hyperparameters

We performed our experiments on a Windows 10 machine with an Intel Core i7-6560U CPU at 2.21 GHz and 16 GB of RAM. We utilized the PyTorch deep learning framework and the Python Python 3.12.7 programming language in all our experiments. A wide experimental effort was invested in tuning various hyperparameters for the best performance. We employed a grid search algorithm to find the best batch size and learning rate, as detailed in Section 4.6 (**Hyperparameter Optimization**). Table 3 depicts the final hyperparameters that were selected and used consistently across all experiments.

These hyperparameters were chosen to ensure training stability, improve generalization, and maintain computational efficacy during the training and evaluation phases.

### 4.3. Models’ Evaluation and Selection for the Proposed Model

In this work, we conducted a comprehensive evaluation of several cutting-edge deep learning models on two independent chest X-ray datasets (dataset 1 and dataset 2). The evaluation was conducted using both predictive performance (accuracy, precision, recall, and F1-score) and computational efficiency (parameters and FLOPs). The results are shown in Table 4.

EfficientNet-B7 showed the best-performing model in both datasets, with 99.14% and 98.75% accuracy in dataset 1 and dataset 2, respectively. Its F1-scores (99.11% and 98.73%) also confirm its excellent performance in detecting TB. However, its model complexity (63.79 million parameters and over 5344M FLOPs) makes it impractical to be used in environments with limited resources. In comparison, MobileViT achieved excellent accuracy (98.48% and 98.75%) at extremely low computational expense (1.94M parameters and 227.91M FLOPs). It also achieved high recall (98.65% and 99.17%) and F1-score (98.46% and 98.76%) across both datasets, which indicates excellent generalization performance. GhostNet also achieved competitive performance with around 95% accuracy, while maintaining the lowest computational expense (3.9M parameters, 52.32M FLOPs), which is ideal for light applications. Other models like VGG-16, ResNet50, and DenseNet-121 also provided high accuracy and F1-scores, but at significantly higher computational costs. For instance, VGG-16 had over 5066M FLOPs, making it unsuitable for real-time or embedded systems.

These outcomes justify our choice of using the combination of GhostNet and MobileViT as the backbone networks in the designed hybrid model.

### 4.4. Performance Analysis of the Proposed Hybrid Model

After selecting the best two models (MobileViT and GhostNet) on the premise of having an ideal trade-off between accuracy and computation efficacy, we developed and tested a novel hybrid model integrating both backbones. We evaluated the proposed model’s performance using two chest X-ray datasets (dataset 1 and dataset 2), and compared it to its individual constituents, as illustrated in Table 5.

The hybrid model outperformed GhostNet and MobileViT on all metrics in both datasets. Specifically, on dataset 1, the hybrid model achieved the highest accuracy (99.52%), precision (99.61%), recall (99.42%), and F1-score (99.51%), demonstrating outstanding detection power with minimal false positives and false negatives. This performance eclipses MobileViT (accuracy of 98.48%, recall of 98.65%) and GhostNet (95.90% accuracy, 95.04% recall), proving the effectiveness of feature fusion between the CNN- and transformer-based branches. On dataset 2, the hybrid model again performed superb generalization with 99.17% accuracy, 99.2% precision, 99.2% recall, and 99.2% F1-score. These outcomes greatly outperformed GhostNet’s poorer performance (94.98% accuracy, 94.02% recall) on the same dataset and beyond MobileViT’s already impressive performance (98.75% accuracy, 99.17% recall).

Computational cost-wise, the hybrid model maintained a fair level of computational complexity, with 7.73 million parameters and 282.11 million FLOPs, which is somewhat higher than MobileViT but still significantly lower than heavier models such as VGG-16 or EfficientNet-B7. This makes the hybrid model both viable and resilient for real-time and resource-limited deployment without sacrificing classification accuracy. In general, the suggested hybrid model successfully combines GhostNet’s efficiency in convolutional processing and MobileViT’s global attention mechanism to create a robust architecture with high-accuracy TB detection across diverse datasets.

**Figure 13:**
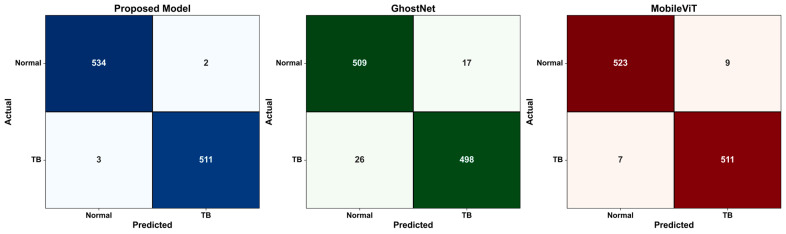
Confusion matrix for the proposed model, GhostNet, and MobileViT on dataset 1.

**Figure 14:**
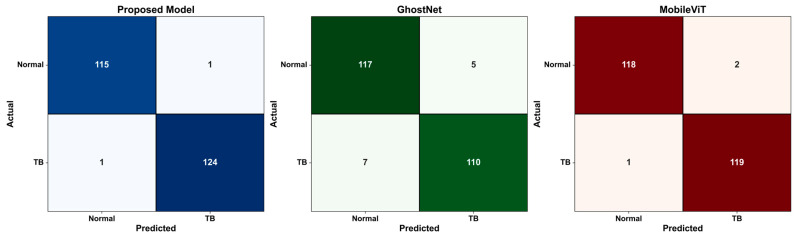
Confusion matrix for the proposed model, GhostNet, and MobileViT on dataset 2.

In order to gain a better insight into each model’s classification accuracy, confusion matrices for dataset 1 and dataset 2 were generated, as shown in Figure 13 and Figure 14. It is noted from these figures that the number of misclassified samples generated by the suggested model for dataset 1 are 5 (2 false positives and 3 false negatives) and 2 (1 false positive and 1 false negative) for dataset 2, where the possibility of this misclassification for the TB images that were classified as normal may lie in that TB lesions are in an early stage. As for the misclassification of the normal images that were classified as TB, the possibility of this misclassification might be due to the fact that these images contain non-TB abnormalities, such as minor infiltrates or artifacts, and based on that, the model classified them as TB-related.

Figure 13 and Figure 14 demonstrate that the suggested hybrid model lowers the possibility of false negatives (TB that goes undetected) in crucial clinical situations by guaranteeing a more balanced and consistent prediction across the normal and TB classes, in addition to providing excellent accuracy.

**Figure 15:**
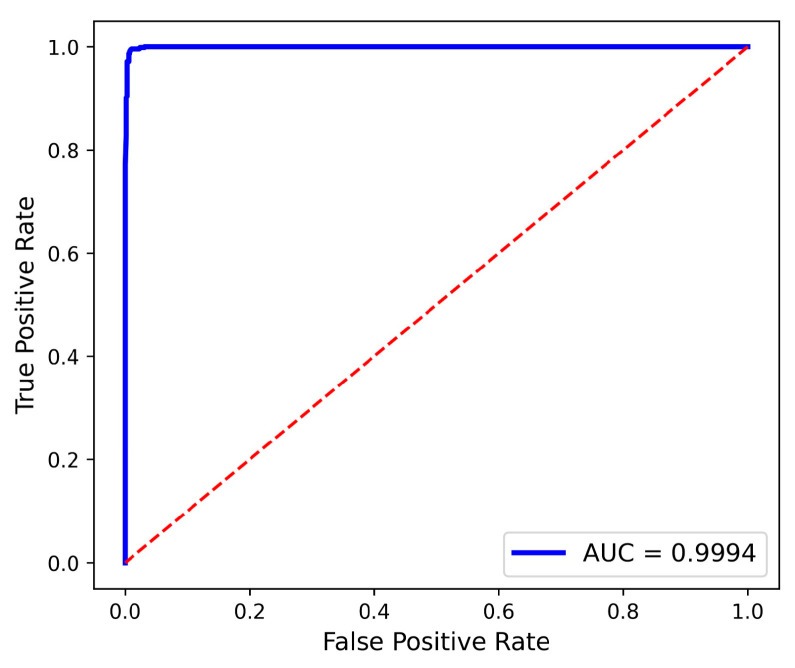
Proposed model ROC curve—dataset 1.

**Figure 16:**
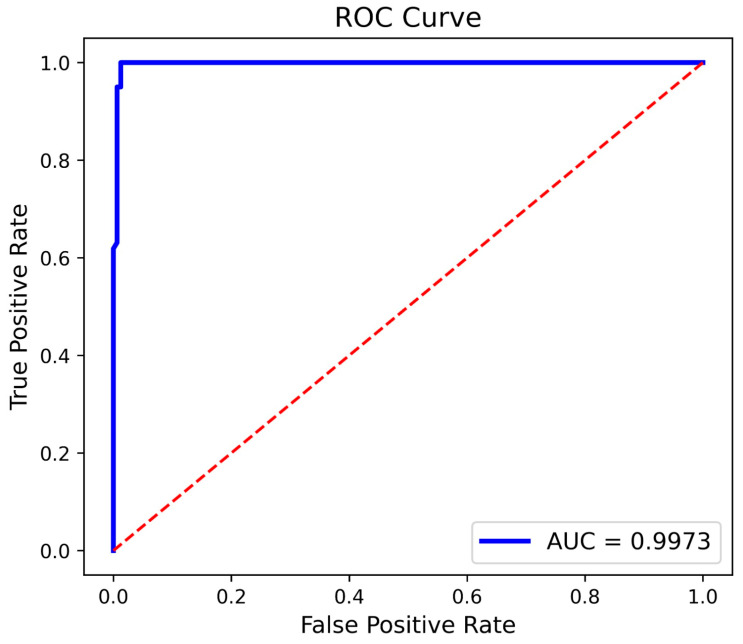
Proposed model ROC curve—dataset 2.

Figure 15 and Figure 16 depict the proposed model’s ROC curve using dataset 1 and dataset 2, respectively.

**Figure 17:**
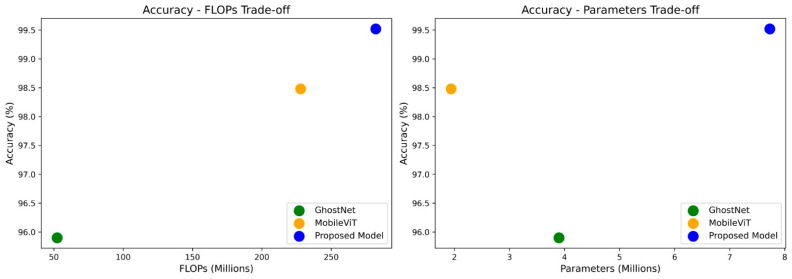
Accuracy–complexity trade-off for dataset1.

**Figure 18:**
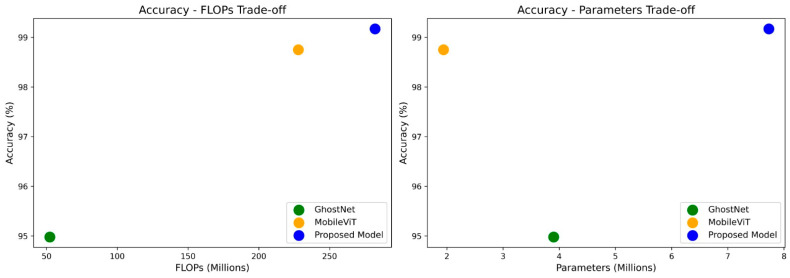
Accuracy–complexity trade-off for dataset2.

Figure 17 and Figure 18 illustrate the accuracy–complexity trade-off for dataset 1 and dataset 2, respectively.

Although the suggested hybrid model has two light-weight structures (GhostNet and MobileViT), it is noted that the overall number of parameters and FLOPs (7.73M and 282.11M) are marginally greater than the expected sum of their individual values (5.84M and 280.23M), as shown in Table 6. This difference is intentional and anticipated, due to the additional layers introduced in the fusion process.

While the backbone models are frozen or pruned to remove redundancy (e.g., by removing BatchNorm layers), the hybrid model combines (1) two additional fully connected layers (256 neurons + output layer); (2) dropout regularization; and (3) a concatenation layer that combines high-dimensional feature maps from both models. These extra layers are not heavy but contribute to the overall complexity, as they are applied on the combined feature vector of size 7424 (1280 from GhostNet + 6144 from MobileViT). Accordingly, the compact increase in FLOPs and parameters has been offset by the outstanding improvement in performance (e.g., F1-Score up to 99.51% on dataset 1 and 99.2% on dataset 2) while continuing to have a significantly smaller computing footprint than large-scale models such as ResNet50 or EfficientNet-B7.

**Figure 19:**
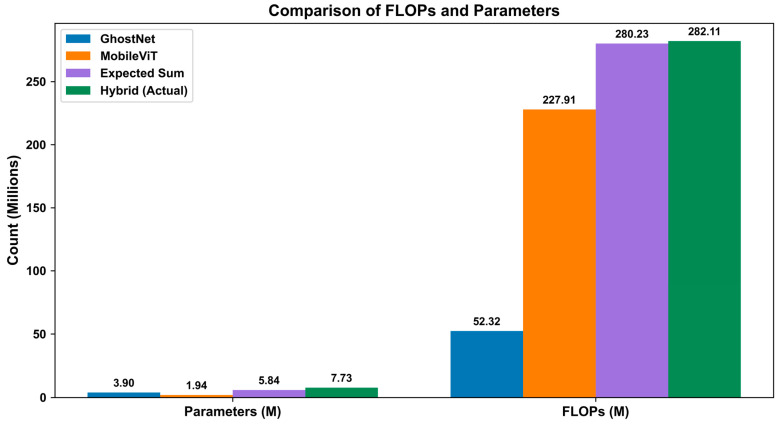
Actual versus expected FLOPs and parameters for the suggested hybrid model.

Figure 19 depicts the actual and expected value of both FLOPs and parameters for the suggested hybrid model.

### 4.5. Additional Validation via 5-Fold Cross-Validation

We performed an additional 5-fold cross-validation mechanism in order to ensure a more robust and statistically reliable evaluation for the suggested model. The dataset has been randomly divided into five mutually exclusive folds. In each iteration, four folds were used for training and validation while the remaining fold was used as the test set. The 5-fold cross-validation performances for dataset 1 and dataset 2 are depicted in Table 7 and Table 8, respectively. The model shows very stable performance across folds with very small standard deviations (SD), hence indicating robust generalization consistency. Moreover, the confidence intervals (CI) at 95% further validate the reliability of the estimates.

### 4.6. Hyperparameter Optimization

To avoid arbitrary selection of hyperparameters, a grid search algorithm has been used to identify the optimal learning rate and batch size for the proposed hybrid model. The search analyzes the performance of combinations of three learning rates (0.0001,0.0005,0.001) and three batch sizes (16,32,64) evaluated on dataset 1 and dataset 2.

Table 9 shows that the combination of batch size = 32 and learning rate = 0.001 achieved the highest performance on dataset 1 (99.53%) and dataset 2 (99.19%); based on that, this configuration was selected for all final experiments.

To verify the effectiveness of the proposed hybrid model, a comprehensive comparison was conducted with several state-of-the-art methods reported in the literature, as presented in Table 10. The table indicates that several previous research employed single DL models, such as CNN [18,19,21], DenseNet201 [39], MobileNet [40], and DenseNet121 [41], which, although achieving reasonable accuracy rates, ranging from 86% to 98.8%, relied on single-model dependency. This reliance on a single model may restrict the system’s ability to capture a broader range of crucial features, potentially reducing robustness and generalizability across diverse clinical scenarios. In addition, many of the works [18,19,49,53,80] evaluated their approach based on small dataset sizes (e.g., Montgomery and Shenzhen), reducing confidence in model scalability and reliability. Several of the proposed models like DenseNet201 [39], ResNet50 [72], and CBAM/WNet [44] attained high efficiency but at the expense of high computational cost making them less preferable for deployment in resource-constrained environments.

The suggested hybrid model, on the other hand, which combines GhostNet and MobileViT, performs better on both Datasets 1 and 2, accomplishing 99.52% accuracy in dataset 1 and 99.17% accuracy in dataset 2, precision of 99.61% in dataset 1 and 99.2% in dataset 2, recall rates of 99.42% for dataset 1 and 99.2% for dataset 2, and an F1-score of 99.2% for dataset 2 and 99.51% for dataset 1. Despite the efficiency boost, the proposed model remains of modest computational complexity, with only 7.73M parameters and 282.11M FLOPs, a minuscule percentage of the cost required by models like EfficientNet-B7 or VGG16, while still achieving state-of-the-art performance. Furthermore, more significantly, even compared to contemporary works utilizing ensemble or stacked classifiers [53], the hybrid model performs equally or even better while being more lightweight and easy to deploy. Briefly, the proposed hybrid approach not only surpasses traditional models in both classification accuracy and robustness but also reaches an even trade-off between performance and efficiency, and thus is a promising candidate for clinical applications.

## 5. Conclusions and Future Works

This paper presented a novel hybrid deep learning model for the detection of tuberculosis from chest X-ray images by integrating GhostNet and MobileViT architectures. The aims was to combine the powers of convolutional neural networks and transformer-based models in order to improve classification accuracy as well as computational efficiency.

To validate the effectiveness of the introduced approach, large-scale experiments have been conducted on two independent chest X-ray datasets. The model was compared to a range of state-of-the-art CNN-based architectures such as VGG-16, ResNet50, DenseNet-121, MobileNetV3, and EfficientNet-B7. Overall, the suggested hybrid model outperformed all baseline models on both benchmark datasets, with results of 99.52% accuracy, 99.61% precision, and 99.51% F1-score on dataset 1 and 99.17% accuracy, 99.2% precision, and 99.2% F1-score on dataset 2. In addition to its terrific predictive efficiency, the hybrid model also retained a lightweight structure, with only 7.73 million parameters and 282.11 million FLOPs, significantly lower than other high-accuracy models like EfficientNet-B7 and VGG-16. Such an efficiency–accuracy balance makes the hybrid model appropriate for real-time clinical use and resource-constrained settings. The efficient combination of GhostNet’s lightweight convolutional architecture and MobileViT’s global feature capturing abilities demonstrates the capacity of multi-branch feature fusion in medical image analysis. Furthermore, the model’s generalizability across multiple datasets confirms its potential as a trustworthy tool in TB screening procedures. Despite such achievements, there are still some areas for future improvement:

• **Multi-Class Classification:** Expanding the binary classification (TB vs. normal) to include several pulmonary diseases, such as pneumonia, fibrosis, or lung cancer. This will make the model more clinically useful.
• **Clinical Deployment and Real-world Validation:** Efforts in the future should also involve testing the model in real clinical settings, its integration with radiology work-flows, and real-time operation on different imaging devices.
• **Cross-Modal Extension:** Investigating hybrid architectures that combine chest X-rays with other modalities (e.g., CT scans or clinical reports) could boost diagnostic performance for difficult or ambiguous cases.
• **External Validation:** To further assess the robustness of the proposed model and its clinical applicability, in future work, we will focus on validating the model using larger, multi-center datasets.

Through these enhancements, the suggested hybrid model can evolve into a robust and clinically powerful platform for AI-assisted TB screening and diagnosis.

## Figures and Tables

**Table 1:** Summary of the related works.

References	Dataset	Methods	Results	Limitations
[33]	Montgomery, Shenzhen, and JSRT	Inception V-3, SVM, KNN, RF, and NN	NN (average accuracy: 80.45%; F1-score: 81.1%; precision: 81.1%; recall: 81.1%; and AUC: 0.894)	The used dataset is small (138 + 662 + 247). The proposed model’s performance is very low.
[34]	Montgomery, and Shenzhen,	HGF, FOSF, SVM	Montgomery dataset: Accuracy = 95.60%; AUC = 0.95. Shenzhen dataset: Accuracy = 99.40%; AUC = 0.99.	Very limited dataset size (138 + 662). The study used handcrafted shape and texture features that cannot learn complex hierarchical patterns.
[35]	Montgomery, and Shenzhen	Several handcrafted feature extraction methods, SVM	Montgomery dataset: AUC = 87%; Accuracy = 78.3%. Shenzhen dataset: AUC = 90%; Accuracy = 84%.	Very limited dataset size (138 + 662). The study used handcrafted feature extraction methods, which cannot learn complex hierarchical patterns. The proposed model’s performance is very low.
[36]	Shenzhen	HOG KNN	Accuracy: 71.81%	The used dataset is very small (662). The performance of the suggested model is really poor. The research employed handcrafted feature extraction techniques that are incapable of learning intricate hierarchical patterns.
[17]	Shenzhen	Deep CNN	The lossless augmentation method attained an accuracy of 70%, while the Lossy augmentation method attained an accuracy of 64%.	The used dataset is very small (662). The suggested model performance is very low. The study employed a single deep CNN model, which may limit its capacity to extract and focus on the most significant features.
[18]	Montgomery, Shenzhen, Belarus	CNN	Accuracy: 86.2%; AUC: 0.925	Performance is very low. The study used a single model. Very limited dataset size (138 + 662 + 306).
[19]	Montgomery, Shenzhen	B-CNN	Montgomery (Accuracy = 96.42%) Shenzhen (Accuracy = 86.46%)	Very limited dataset size (138 + 662). Performance is relatively low. The study employed a single model.
[20]	Montgomery, Shenzhen, and private dataset (FAHXJU)	Faster RCNN with reinforcement learning	Montgomery dataset: Accuracy = 0.926% Shenzhen dataset: Accuracy = 0.902%	The suggested model performance is relatively low.
[21]	Dhaka-Qatar	CNN	For all classes of diseases (accuracy: 98.72%). For TB detection (precision: 98.9%; recall: 98.1%; F1-Score: 98.5%)	The study relied on a single model, which may lack the capacity to focus on the most crucial features. Did not address the dataset imbalance issue.
[39]	NLM, Belarus, NIAID, and RSNA.	ResNet18, ResNet50, ResNet101, ChexNet, InceptionV3, Vgg19, DenseNet201, SqueezeNet, and MobileNet	DenseNet201(accuracy: 98.6%; precision: 98.57%; sensitivity: 98.56%; F1-score: 98.56; and specificity: 98.54%)	High computational cost. Single-model dependency.
[40]	NIH, Shenzhen, and Montgomery	MobileNet	Overall accuracy: 98.66% For normal class (recall: 98.66%; precision: 99.41%; F1-score: 0.986; specificity: 99.41%). For infected class (recall: 99.42%; precision: 97.93%; F1-score: 0.986; specificity: 97.93%)	Single-model dependency.
[41]	NIAID, and Dhaka-Qatar	DenseNet121	Accuracy: 90%; precision: 92%; recall: 90%; F1-score: 89%; AUC score: 0.976	Performance is relatively low. High computational cost. The study relied on a single model.
[42]	Montgomery, Shenzhen, and a private dataset (Thailand)	AlexNet, VGG-16, CapsNet	CapsNet (accuracy: 80.06%; sensitivity: 92.72%)	Low performance. Single-model dependency
[43]	Montgomery, and Shenzhen,	VGG16	Accuracy without augmentation: 80%. Accuracy with data augmentation: 81.25%.	Very limited dataset size (138 + 662). Very low performance. The study relied on a single model.
[44]	Montgomery, Shenzhen, and Dhaka-Qatar	CBAM, WDnet	Accuracy: 98.80%; sensitivity: 94.28%; precision: 98.50%. Specificity: 95.7%, F1-score: 96.35%.	The model is computationally intensive.
[45]	Montgomery, and Shenzhen	ResNet with a simple external attention mechanism	Accuracy: 97.59%; sensitivity: 100%; AUC: 97.8%; specificity: 95.56%; precision: 95%.	The used dataset is very small (138 + 662). The proposed model is a shallow network that could struggle to discern the complex and hierarchical patterns in very large or heterogeneous datasets.
[46]	Shenzhen	ResNet and EfficientNet	Accuracy: 89.92%; AUC: 94.8%.	The used dataset is very small (662). The suggested model performance is relatively low
[47]	Montgomery, Shenzhen, Kenya, and India	AlexNet, VGG-16, VGG-19, Xception, ResNet-50	Shenzhen: Best accuracy 85.5% (VGG-16). Montgomery: Best accuracy 75.8% (Xception). Kenya: Best accuracy 69.5% (AlexNet, VGG-16). India: Best accuracy 87.6% (VGG-16).	Single-model dependency: Although multiple models were evaluated, the study ultimately relied on a single model for classification, which may restrict the system’s ability to capture a broader range of crucial features. The suggested model performance is relatively low
[48]	Shenzhen	MobileNet, AEO	Accuracy = 90.2%	The utilized dataset is very small (662). The suggested model performance is relatively low.
[49]	Montgomery, Shenzhen, and Belarus	CNN, LSTM	Accuracy: 96.26%; precision: 96.44%; recall: 96.62%; and F1-score: 96.49%.	Very limited dataset size (138 + 662 + 306). The study did not address the dataset imbalance issue.
[50]	Montgomery and Shenzhen	ResNet–SVM	Accuracy: 93.91%; precision: 93%; and AUC: 91%.	Very small dataset (138 + 662). The suggested model performance is relatively low.
[51]	NIAID, NLM, and Belarus	CNN, RNN, ANN	Accuracy: 97%; precision: 85%; recall: 90%; F1-score: 88%.	The precision, recall, and F1-score metrics are low. The suggested hybrid model, integrating CNNs, ANN, and RNN, increases computational complexity.
[52]	Belarus, Montgomery, Shenzhen, and JSRT	Ensemble technique (AlexNet, GoogleNet, and ResNet)	Accuracy: 88.24%; sensitivity: 88.42%; specificity: 88%; AUC: 0.93%.	The performance of the suggested model is low. High computational cost.
[53]	Montgomery, and Shenzhen	Xception, DenseNet, and Stacked ensemble classifier (LR, DT, RF, SVM, and AdaBoost)	Shenzhen (Specificity: 99.47%, Sensitivity: 99.39%, AUC: 0.98, and Accuracy: 99.22%). Montgomery (Specificity: 99.15%, Sensitivity: 99.42%, AUC: 0.99, and Accuracy: 99.26%)	Very small dataset (138 + 662). High computational cost.
[54]	Montgomery, Shenzhen, Kenya, and India	Several handcrafted feature extraction methods (HOG, GIST, and SURF), several pre-trained CNN algorithms (AlexNet, VGG-16, GoogLeNet, ResNet-50), and SVM.	Shenzhen (Accuracy: 93.4%), Montgomery (Accuracy: 87.5%), Kenya (Accuracy: 77.6%), and India (Accuracy: 96.0%).	The performance of the suggested model is relatively low. High computational cost.

**Table 2:** Summary of dataset characteristics.

Dataset	Dataset 1	Dataset 2
Source	CXR database [39]	TBX11K dataset [55]
Total Images	7000	1600
TB Cases	3500	800
Normal Cases	3500	800
Training (70%)	4900	1120
Validation (15%)	1050	240
Testing (15%)	1050	240

**Table 3:** Summary of the optimized hyperparameter.

Hyperparameter	Value
Learning Rate	0.001
Optimizer	Adam
Loss Function	Cross-Entropy Loss
Batch Size	32
Number of Epochs	10
Dropout Rate	0.5

**Table 4:** Performance evaluation of several cutting-edge deep learning models.

	Model	Accuracy (%)	Precision (%)	Recall (%)	F1-Score (%)	Parameters (M)	FLOPs (M)
**Dataset 1**	GhostNet	95.90	96.70	95.04	95.86	3.9	52.32
	MobileViT	98.48	98.27	98.65	98.46	1.94	227.91
	VGG-16	96	96.56	95.46	96.01	52.48	5066.59
	DenseNet-121	96.29	95.28	97.3	96.28	6.96	945.63
	MobileNetV3	97.62	96.3	98.27	97.77	4.2	78.11
	ResNet50	98.29	98.11	98.48	98.3	23.51	1349.13
	EfficientNet-B7	99.14	99.56	98.24	99.11	63.79	5344.54
**Dataset 2**	GhostNet	94.98	95.65	94.02	94.78	3.9	52.32
	MobileViT	98.75	98.35	99.17	98.76	1.94	227.91
	VGG-16	97.08	98.15	96.12	97.1	52.48	5066.59
	DenseNet-121	95.83	95.43	97.22	96.24	6.96	945.63
	MobileNetV3	98.33	98.2	98.64	98.41	4.2	78.11
	ResNet50	96.67	96.16	97.33	96.72	23.51	1349.13
	EfficientNet-B7	98.75	98.32	99.15	98.73	63.79	5344.54

**Table 5:** Performance evaluation of the suggested model.

	Model	Accuracy (%)	Precision (%)	Recall (%)	F1-Score (%)	Parameters (M)	FLOPs (M)	Inference Time (MS)
**Dataset 1**	GhostNet	95.90	96.70	95.04	95.86	3.9	52.32	33.01
	MobileViT	98.48	98.27	98.65	98.46	1.94	227.91	27.24
	Proposed Model	99.52	99.61	99.42	99.51	7.73	282.11	65.28
**Dataset 2**	GhostNet	94.98	95.65	94.02	94.78	3.9	52.32	34.68
	MobileViT	98.75	98.35	99.17	98.76	1.94	227.91	29.53
	Proposed Model	99.17	99.2	99.2	99.2	7.73	282.11	66.96

**Table 6:** Actual vs. expected FLOPs and parameters for the proposed hybrid model.

Metric	GhostNet	MobileViT	Sum (Expected)	Hybrid (Actual)
Parameters (M)	3.90	1.94	5.84	7.73
FLOPs (M)	52.32	227.91	280.23	282.11

**Table 7:** Five-fold cross-validation performances for dataset 1.

Fold	Accuracy (%)	Precision (%)	Recall (%)	F1-Score (%)
1	99.57	99.71	99.43	99.57
2	98.79	98.03	99.57	98.8
3	99.43	99.43	99.43	99.43
4	99.93	99.88	99.86	99.93
5	99.553	99.62	99.42	99.52
**Mean ± SD**	99.45 ± 0.37	99.33 ± 0.67	99.54 ± 0.17	99.45 ± 0.37
**95% CI:**	[98.99, 99.92]	[98.50, 100.16]	[99.33, 99.75]	[98.99, 99.91]

**Table 8:** Five-fold cross-validation performances for dataset 2.

Fold	Accuracy (%)	Precision (%)	Recall (%)	F1-Score (%)
1	99.38	98.77	99.98	99.38
2	98.75	98.93	97.5	98.73
3	99.15	99.17	99.29	99.16
4	99.89	99.91	99.93	99.88
5	98.56	98.73	99.17	98.56
**Mean ± SD**	99.15 ± 0.47	99.10 ± 0.43	99.17 ± 0.90	99.14 ± 0.47
**95% CI:**	[98.56, 99.73]	[98.56, 99.64]	[98.06, 100.29]	[98.56, 99.73]

**Table 9:** Grid search results for hyperparameter optimization.

	Learning Rate	Batch Size	Accuracy (%)
**Dataset 1**	16	0.0001	98.86
		0.0005	97.43
		0.001	98.95
	**32**	0.0001	98.57
		0.0005	99.24
		**0.001**	**99.53**
	64	0.0001	99.14
		0.0005	98.38
		0.001	98.29
**Dataset 2**	16	0.0001	97.92
		0.0005	97.08
		0.001	99.01
	**32**	0.0001	99.12
		0.0005	99.19
		**0.001**	**99.19**
	64	0.0001	97.92
		0.0005	98.75
		0.001	97.5

**Table 10:** Comparison of the suggested method against previous state-of-the-art methods.

Reference	Dataset	Methods	Results
[18]	Montgomery, Shenzhen, Belarus	CNN	Accuracy: 86.2%
[19]	Montgomery, Shenzhen	B-CNN	Montgomery (Accuracy = 96.42%). Shenzhen (Accuracy = 86.46%)
[21]	Dhaka-Qatar	CNN	Accuracy: 98.72% Precision: 98.9% Recall: 98.1% F1-score: 98.5%
[39]	NLM, Belarus, NIAID, and RSNA.	DenseNet201	Accuracy:98.6% precision: 98.57% F1-score: 98.56
[40]	NIH, Shenzhen, Montgomery	MobileNet	Overall accuracy: 98.66%
[41]	NIAID, Dhaka-Qatar	DenseNet121	Accuracy: 90% Precision: 92% Recall: 90% F1-score: 89%
[44]	Montgomery, Shenzhen, and Dhaka-Qatar	CBAM, WDnet	Accuracy: 98.80% Precision: 98.50% F1-score: 96.35%
[49]	Montgomery, Shenzhen, and Belarus	CNN, LSTM	Accuracy: 96.26% precision: 96.44% Recall: 96.62% F1-score: 96.49%
[80]	Montgomery, Shenzhen	VGG16, Bi-LSTM	Shenzhen (Accuracy: 97.76%). Montgomery (accuracy: 96.42%)
[53]	Montgomery, Shenzhen	Xception, DenseNet, and Stacked ensemble classifier (LR, DT, RF, SVM, and AdaBoost)	Shenzhen (Accuracy: 99.22%). Montgomery (Accuracy: 99.26%)
[72]	CXR dataset (dataset 1)	ResNet50	Accuracy: 99.2% F1-score: 99.18%
This study	Dataset 1	Hybrid model (GhostNet and MobileViT)	Accuracy: 99.52% Precision: 99.61% Recall: 99.42% F1-score: 99.51% Params: 7.73 FLOPs: 282.11
This study	Dataset 2	Hybrid model (GhostNet and MobileViT)	Accuracy: 99.17% Precision: 99.2% Recall: 99.2% F1-score: 99.2% Params: 7.73 FLOPs: 282.11

## Data Availability

All used datasets were obtained from open sources. The reader can refer to [40,61] in this paper. The source code is available upon request from the corresponding author.
